# Improved Monitoring of *Grapholita molesta* (Lepidoptera: Tortricidae) in Stone Fruit Orchards with a Pheromone-Kairomone Combination Lure

**DOI:** 10.3390/insects11070412

**Published:** 2020-07-03

**Authors:** Michele Preti, Alan L. Knight, Sergio Angeli

**Affiliations:** 1 Faculty of Science and Technology, Free University of Bozen-Bolzano, piazza Università 5, 39100 Bolzano, Italy; michele.preti@natec.unibz.it; 2 Instar Biologicals, Yakima, WA 98908, USA; uncfencer76@hotmail.com

**Keywords:** oriental fruit moth, sex pheromone, monitoring, trap, mating disruption, terpinyl acetate, acetic acid

## Abstract

Monitoring oriental fruit moth *Grapholita molesta* Busck (Lepidoptera: Tortricidae), populations based on counts in sex pheromone-baited traps within sex pheromone-treated orchards for mating disruption (MD) is challenging since male orientation to traps is disrupted. In this study, we evaluated a new commercial pheromone–kairomone combination lure for *G. molesta* (Pherocon^®^ OFM Combo™ Dual™), which combines the *G. molesta* and *Cydia pomonella* L. sex pheromones with a blend of acetic acid and terpinyl acetate. Trap comparisons were performed in 33 trials in peach and nectarine orchards under MD (24) or non-MD (9) during the 2018–2019 period in Italy. Male and total moth captures in traps baited with the combination lure were significantly greater than in traps with *G. molesta* pheromone in both years and across both pheromone treatments. The proportion of females in the total moth captures using the combination lure averaged ca. 7% across all trials. The by-catch of non-targets, i.e., *Apis mellifera* L., was low in traps baited either with the combination and the sex pheromone lure, while trap color (white) affected the capture of beneficials but not of the target pest. Our study proves that this combination lure can improve the monitoring and management for *G. molesta* in stone fruits. New studies are needed to define action thresholds to trigger supplemental control methods to MD. Secondly, female-based monitoring lures should be further developed to improve management strategies.

## 1. Introduction

The oriental fruit moth, *Grapholita molesta* Busck (Lepidoptera: Tortricidae), is a global key pest of peaches and nectarines (*Prunus persica* L.), as well as an important pest of other stone and pome fruit crops such as plums (*Prunus salicina* Lindl.), apricots (*Prunus armeniaca* L.), apples (*Malus domestica* Borkh.) and pears (*Pyrus communis* L.) [1]. In many countries, peach and nectarine orchards are typically managed under Integrated Fruit Production (IFP) plans, which include the use of sex pheromones for the Mating Disruption (MD) of *G. molesta*, together with some supplemental insecticidal applications according to the forecasting models for pest occurrence. In Italy, MD was first applied for *G. molesta* in the early 1990s and it was largely adopted from the year 2000 onward [2], covering most of the Italian peach and nectarine orchards to date in an area-wide program, as well as in other countries [3,4]. 

In MD-treated orchards, monitoring the adult pest population is particularly challenging using only sex pheromone-baited traps, since the male moths may not be able to locate the monitoring traps and thus get captured. In fact, due to the high sex pheromone concentration in the environment using MD, to the reduced response of pheromone-exposed male moths and to the competitive attraction between the natural and synthetic sex stimulus, the insects are not able to properly orientate towards the odor source [5,6]. In particular, field studies have demonstrated that the male behavior varies according to the amount of sex pheromone loaded and released by the MD dispensers (in relation to the characteristics and density of the pheromone emitters), resulting in the males following a false-plume and therefore being “sexually distracted” rather than creating a proper “sexual disruption or confusion” with no male orientation [7]. To overcome this problem, growers typically place the sex pheromone-baited traps in the edges of their MD-treated orchards in the hope to intercept male flight activity. Despite that, monitoring the adult moths with such traps is not effective, providing poor and inconsistent information about the insect pest infestation. In addition, growers commonly assess the pest occurrence in MD-treated orchards through a labor-intensive visual sampling of the plant canopy, detecting larval shoot and fruit injury and adjusting the timing of insecticidal spray with forecasting models. The use of supplemental insecticidal sprays under MD is usually the consequence of high pest pressure, or when using edge located traps to determine pest levels, since it is possible to observe “edge-effect” on pest occurrence in MD-treated orchards [8]. Recently, various *G. molesta* infestations have occurred in MD-treated peach and nectarine orchards in the Emilia-Romagna region (Italy) [9], where growers usually determine their control intervention timing based on forecasting models. 

Over the past 20 years, several studies have been conducted to develop alternative monitoring methods. These have included the identification and testing of various kairomones, specifically host plant Volatile Organic Compounds (VOCs) attractive to both *G. molesta* sexes. For instance, in 2003, Natale [10] tested synthetic blends of (*Z*)-3-hexen-1-yl acetate, (*Z*)-3-hexen-1-ol and benzaldehyde, while Piñero and Dorn [11] tested the same VOCs in combination with other compounds such as (*E*)-2-hexenal and benzonitrile, both showing an attractiveness of these blends for *G. molesta* female moths. In 2009, Il’ichev et al. [12] demonstrated that males of *G. molesta* are also attracted by host plant VOCs such as (*Z*)-3-hexen-1-yl acetate, (*E*)-β-ocimene and (*E*)-β-farnesene. More recently, a synergistic effect between kairomone and pheromone compounds has been demonstrated testing mixtures of these host plant VOCs combined with the female sex pheromone [13,14]. 

Liquid food bait traps have also been investigated, for instance unique traps baited with terpinyl acetate and brown sugar have been developed to capture both sexes of *G. molesta* [15,16,17]. Recent studies have shown that a combination of codling moth *Cydia pomonella* L. sex pheromone ((*E,E*)-8,10-dodecadien-1-ol, codlemone) and the three main components of the *G. molesta* sex pheromone ((*E*)-8-dodecen-1-yl-acetate, (*Z*)-8-dodecen-1-yl acetate and (*Z*)-8-dodecen-1-ol) enhanced *G. molesta* captures [18], investigating acetic acid as a synergistic compound, also [18,19]. Finally, in 2018, Mujica et al. [20] reported a very interesting dual-sex attractiveness of several experimental combinations of the above-mentioned pheromone blends synergized by terpinyl acetate and acetic acid. 

The present study aimed to evaluate the efficacy of a new combination lure, Pherocon^®^ OFM Combo™ Dual™ (industrialized by Trécé Inc., Adair, OK, USA and commercially available since 2018), to monitor *G. molesta* trap captures in MD-treated and non-MD-treated peach and nectarine orchards. This dry binary lure is composed of a *G. molesta* and *C. pomonella* sex pheromones combination in a rubber septum plus acetic acid and terpinyl acetate loaded together in a membrane cup. 

## 2. Materials and Methods

Thirty-three field trials comparing lures were carried out in peach and nectarine orchards located in the Emilia-Romagna region (Italy) over a two-year period in 2018–2019. In 2018, 13 trials were performed in MD-treated orchards, while in 2019, 11 and 9 trials were performed in orchards treated and not treated with MD, respectively. Trial locations were selected from sites with a previous high pest pressure of *G. molesta* and where growers signaled larval damage (either on shoots or both on shoots and fruits). The trials were conducted between March and October to include the different seasonal periods of the crop, according to the BBCH crop phenological scale (used to identify the plant phenological development stages) [21], to capture moths during the entire *G. molesta* flight period. The trials included cultivars that are harvested between early June and mid-September. Details of the trial execution in relation to the crop phenology of the selected cultivars, according to [22], are reported in Table 1.

Some orchards were utilized for more than one trial during different seasonal periods, as reported in Table 2. A variety of MD application methods including passive dispensers, sprayables, and aerosol emitters were present in the MD-treated orchards under study. More complete details of trial locations and MD information are listed in Table 2.

All trials compared two lures: the new combination lure Pherocon^®^ OFM Combo Dual and the classical *G. molesta* sex pheromone lure Pherocon^®^ OFM L^2^ (both from Trécé Inc.). The combination lure was delivered in a grey halobutyl septum loaded with (*E*)-8-dodecen-1-yl-acetate, (*Z*)-8-dodecen-1-yl acetate, (*Z*)-8-dodecen-1-ol and codlemone to be used in combination with a proprietary membrane cup loaded with a mixture of acetic acid and terpinyl acetate. The *G. molesta* sex pheromone lure was a grey halobutyl septum loaded with (*E*)-8-dodecen-1-yl-acetate, (*Z*)-8-dodecen-1-yl acetate and (*Z*)-8-dodecen-1-ol. 

A standardized protocol was used in both years to compare captures. Delta-shaped traps with wire hangers (Pherocon^®^ VI, Trécé Inc.) were baited with either the combination or the sex pheromone lure. Preliminary trials showed that unbaited traps did not catch any moths. Trials in 2018 used either white or orange delta traps, while only white delta traps were used in 2019 (Table 1). These two trap colors were selected in 2018 since they are the most used among the growers, while in 2019, considering the results of the first year on beneficials, the researchers focused the experiments only on white traps as “worst case” of selectivity. In each trial, five or six replicates of each lure type were randomized within the orchard and spaced at least 25 m from the orchard’s perimeter and apart (Table 1). Traps were placed at a height of ca. 1.8 m. Traps were checked weekly, captures were counted and sexed, and liners were replaced. The trial duration was 6-8 weeks, equal to the lure’s longevity.

Statistical analyses of the captures (males, females, and total *G. molesta*) cumulated over the trial period were performed with R software (v. 4.0.0, R Core Team 2020) [23]. 

The comprehensive analyses of data captures recorded from different trials were performed with a Generalized Linear Mixed Models using Template Model Builder (glmmTMB) [24]. After comparing deviance and Akaike’s information criteria (AIC) for fitted glmmTMB with different distributions, the capture data were found to be non-normally distributed (given the nature of the count data in our study, with numerous zero captures), fitting best with a negative binomial distribution. Captures in 2018 and 2019 trials were analyzed separately and in 2019 the trials performed in MD and non-MD orchards were analyzed separately. The effect of the year was evaluated in the model selecting for both years only the trials performed within MD-treated orchards. The effect of MD management was evaluated only for trials conducted in 2019. Since the trials were repeated across different orchards and dates, the trial number, location (i.e., farm), crop cultivar and trial start date were set as random effects in the model. To fit the model, the fifteen cultivars were also grouped in two categories (peach and nectarines), while the numerous trial start dates were also grouped in three categories (early fruit development, late fruit development and post-harvest). Together with the lure treatment, also these two factors (i.e., crop type and crop phenology) were considered as predictive variables. 

Mean data within each trial were compared using unpaired two-sample *T*-test, data were checked for normality using Shapiro–Wilk test and equal variance using an F-test. Count data were transformed prior to analysis with a square-root and a *p*-value of 0.05 was used to establish significance in all tests. When data from single trials were not normally distributed the non-parametric unpaired two-sample Mann–Whitney U test was adopted (*p*-value at 0.05). Single-trial analyses were performed only when both treatments accounted captures (i.e., only for male captures in 21 trials), while when captures were present in only one treatment (e.g., female captures were zero in all the sex pheromone-baited traps) the single-trial analysis was not performed.

Additional comprehensive analyses were conducted with the 2018 data including the effect of trap color and lure type on both the captures of the target pest (*G. molesta*) and of beneficials such as honeybees (*Apis mellifera* L.), while 2019 data were analyzed to evaluate lure selectivity towards honeybees using only white colored traps. Data of other non-target insect species such as dipterans (Diptera: Muscidae) were not analyzed due to the very low and not consistent values recorded across all the study. *Z* and *p* value were considered as outputs of the model to discriminate significant differences.

## 3. Results

### 3.1. Comprehensive Analyses on G. molesta

The results of the cumulative statistical analyses of *G. molesta* captures across trials are presented in Table 3, separated by year and management type.

In both years, a high significant difference was observed between the two lure treatments in terms of mean male and total *G. molesta* captures, regardless of the MD application. In MD orchards, the total *G. molesta* captures with the combination lure were on average at least three-fold higher (up to 32-fold higher) than the sex pheromone captures, while in non-MD orchards, the difference was 6.5-fold, on average. Over the trial period of six to eight weeks, female captures per trap (± SEM) using the combination lure were on average 0.64 (± 0.17) in 2018 and 1.22 (± 0.21) in 2019 in MD-treated orchards, while 4.71 (± 0.90) in 2019 in orchards not treated with MD (in Table 3, female proportions are reported). 

The capture data across trials were also analyzed to evaluate the effect of year, MD management, crop type and crop phenology on the *G. molesta* captures, as reported in Table 4. The year had not had an effect on the moth captures within MD-treated orchards. On the contrary, using the management as a predictive variable in the model, the MD application revealed to impact the *G. molesta* captures (higher in non-MD orchards than in MD-treated orchards, as visible in Table 3). Crop type had an effect on the moth captures only in 2018 (where the moth flight in peach orchards was lower compared to the moth flight recorded in nectarine orchards), while crop phenology showed an effect on the moth captures only in 2019 (see Table 4). Concerning the moth flight monitoring conducted in 2019 under MD, the trials performed in the late fruit development crop stage and post-harvest had less captures than the trials performed in early fruit development crop stage. In contrast, the moth flight monitoring conducted in 2019 in absence of MD showed the opposite trend: the trials performed in post-harvest had a higher number of moth captures compared to the trials performed in the first part of the season.

### 3.2. Single-Trial Analyses

Single-trial results, grouped by MD usage and year, are reported in Table 5, Table 6 and Table 7 according to the different seasonal periods in which the trials were performed. Considering the trial start–end date and the cultivar harvest date, trials were grouped in: early fruit development when performed between blooming and fruits at half final size (Table 5); late fruit development when performed between fruits at half final size and harvest (Table 6); post-harvest when started after fruit harvesting, therefore when *G. molesta* develops only in the vegetative part of the plants (i.e., on the shoots) (Table 7). The *G. molesta* adult captures were not uniform across the trials, ranging from less than one to more than 100 cumulated captures per trap in the six- to eight-week trial period. In 12 out of 33 trials, only the combination lure-baited traps were able to capture *G. molesta* moths, while zero captures were recorded in the traps baited with the sex pheromone lure. However, some moths were also captured in traps baited with the sex pheromone lure in ca. half of all the trials within MD-treated orchards (8 out of 13 trials in 2018, 5 out of 11 trials in 2019), showing a certain male response to the sex pheromone lure alone also under MD. Considering the trials individually, differences in *G. molesta* captures between the combination and the sex pheromone lure were significant (*p* < 0.05) in almost every trial, with few exceptions, where the two lures provided a similar performance. 

Female moths were only captured in traps baited with the combination lure in any trial, both in MD and non-MD-treated orchards (Table 5, Table 6 and Table 7). The higher numbers of *G. molesta* female captures were recorded in non-MD-treated orchards after fruit collection (up to 17 female moths captured per trap during the trial period) (Table 7). Over both years, in the traps baited using the combination lure, female moth captures accounted, on average, for only 7.3% of the total *G. molesta* captures.

### 3.3. Data Analyses on Non-Target Species and Trap Color Effect

**Figure 1:**
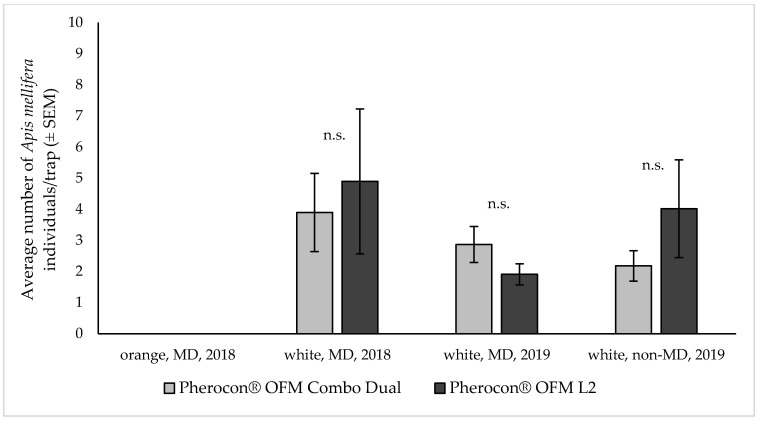
Honeybees (*Apis mellifera* L.) captured by either orange or white colored delta traps baited with lures used to monitor *Grapholita molesta* flight in peach and nectarine orchards. Mean (± SEM) total honeybee captures over the trial period (six to eight weeks) in traps baited with Pherocon^®^ OFM Combo Dual and Pherocon^®^ OFM L^2^ (Trécé Inc.). Trials were performed in orchards treated or not treated with mating disruption (MD and non-MD, respectively): in 2018 n = 13 under MD; in 2019 n = 11 under MD and n = 9 in non-MD orchards. Statistical analysis performed with a Generalized Linear Mixed Models using Template Model Builder (glmmTMB), n.s. = differences not significant.

The trap and lure selectivity were high in both years, with a very low abundance of non-target insects captured. In both years, a few specimens (< four to five units per trap over the trial period) of Diptera (mainly Muscidae), Lepidoptera (mainly Noctuidae) and Hymenoptera (mainly Apoidea) were recorded in some of the trials. In particular, honeybees were captured on the sticky liners but in limited and inconsistent numbers. Considering the importance of beneficials such as honeybees, and since *A. mellifera* was the most abundant species among the non-target captures, a specific analysis on this pollinator was performed to investigate the selectivity of the combination lure in comparison to the sex pheromone lure. On average, ca. three to four honeybee captures were recorded per trap over the whole trial period, regardless of the lure used (Figure 1). In fact, analyzing the honeybee captures, no difference emerged between the two tested lures (df = 124; z = −0.677 and *p* = 0.498 for data collected in 2018 under MD; df = 100; z = −0.970 and *p* = 0.332 for data collected in 2019 under MD; df = 80; z = –0.924 and *p* = 0.356 for data collected in 2019 without MD). In 2018, orange colored traps never captured honeybees, which were captured only during trials using the white colored traps in both 2018 and 2019 (Figure 1). 

Finally, white or orange delta traps were equally attractive to *G. molesta* in 2018. In fact, trap color did not have an effect on the captures of either sex of *G. molesta* (male moths: df = 126, z = −0.811 and *p* = 0.418; female moths: df = 126, z = −1.582 and *p* = 0.114).

## 4. Discussion

In both 2018 and 2019, the combination lure, Pherocon^®^ OFM Combo Dual, outperformed the standard sex pheromone lure. The statistically higher *G. molesta* captures with this dual-sex combination lure observed in our trials was consistent with our previous study, where several new experimental blends including this one were compared in field experiments in the United States, South America and Italy [20]. However, in this study, testing a commercially available lure (in which the load amount of the known compounds is protected by copyright), the female proportion was lower compared to that recorded in Mujica et al. [20], where we tested similar experimental proprietary blends. 

In this study, we captured *G. molesta* from early March through the beginning of October, both in MD- and non-MD-treated peach and nectarine orchards. The moth’s flight activity was successfully monitored throughout the vegetative season with the pheromone–kairomone combination lure, which revealed to be attractive both in the presence and absence of fruits (Table 5, Table 6 and Table 7). As expected, in non-MD orchards, the moth flight recorded was generally higher compared to that observed in MD-treated orchards (Table 3), probably also due to the effect of MD on the *G. molesta* behavior; in addition, in non-MD orchards the moth flight recorded at the end of the season was higher overall compared to that observed in the early season (Table 4), probably due to the lack of grower interventions post-harvest. The reverse trend observed in MD-treated orchards was also in line with expectations, since data captures of trials conducted in early season (adult moths coming from the overwintering populations) were—in general—higher than in trials conducted in the late season (when growers usually apply broad-spectrum insecticides also for other target pests, such as *Halyomorpha halys* Stål) or post-harvest (when growers interrupt the insecticidal sprays, but MD should however be effective). 

The level of adult moth captures differed across the trials and locations and this high variability can be explained with the different history of each orchard, likely due to the present and past grower’s insecticide spray programs, which directly or indirectly have affected the *G. molesta* populations to an unknown degree. However, the level of adult moth captures in the traps does not necessarily have a direct association with the level of larval pest infestation and the consequent crop damage, and a number of factors that affect the capture–damage relationship should be always taken into account [25]. Within our study, we operate in sixteen peach and nectarine orchards with previous histories of *G. molesta* crop damage occurrence, and also, during the 2018–2019 period, the larval damage was detected, although not recorded. Despite the fact that no speculation can be carried out between the adult flight (registered per every trial) and the offspring larval activity (unknown in our study), the adult moth monitoring with the combination lure-baited traps can be juxtaposed with the forecasting model to provide a more complete and useful set of information in order to better target the control timings and decisions. As reported by Preti et al. [9], the presence of shoot or fruit damage and moth flight in MD-treated orchards can have several explanations, but practically, growers with fruit injury are not able to predict when to spray insecticides in MD-treated orchards using only the sex pheromone-baited traps, resulting in a low efficacy and effectiveness of their spray programs against *G. molesta*. This study demonstrated that the combination lure can be a valid and trustworthy tool to replace the sex pheromone for a more effective moth flight monitoring in the presence of MD.

Regarding non-target species, particularly in our study, honeybees were captured, although in limited and inconsistent numbers. The new pheromone–kairomone lure was not more attractive for honeybees compared to the sex pheromone lure, both showing a similar selectivity. Honeybees were captured mainly due to the orchards nearness to apiaries located in the fruit production area selected for this study, especially in the early spring for pollination service during blooming, and only in white colored traps. Previous studies have shown that trap color can impact the capture of beneficials, and the white color in particular affects honeybees but not the target pest captures [26,27]. After observing that in 2018 few beneficials were captured only in white traps, in 2019, we decided to use only white traps (the worst case) to better measure the impact of white colored traps on honeybees using the combination lure: our results show that the non-target capture issue was negligible and the new lure was not more attractive than the standard sex pheromone, suggesting that the trap color had the major effect on non-target captures. We also confirmed that there is not a trap color effect on the target pest (between orange and white colors). 

The attraction of female moths to the combination lure (<10% of the total *G. molesta* captures with this dual-sex lure) can be imputable to the presence of kairomonal compounds (i.e., acetic acid and terpinyl acetate) in the new lure. The female occurrence in traps opens new opportunities for future *G. molesta* female monitoring and management: (*i*) female presence in the orchard could be measured using Pherocon^®^ OFM Combo Dual for a more timely application of control intervention methods targeting eggs and larvae; (*ii*) female mating status and virginity ratio in MD-treated orchards could be performed to assess the MD effectiveness, considering that MD use can reduce multiple matings, as observed by Knight [28] on *C. pomonella*; (*iii*) female removal using non-saturating bucket traps baited with this lure could be attempted, in combination with other control techniques to perform a mass trapping of *G. molesta* as it was described for *C. pomonella* [29].

Further studies should be performed, testing the new combination lure in order to better investigate the *G. molesta* female behavior towards this blend, considering also that female moths can “autodetect” their own sex pheromone [30], which is present in this dual-sex lure and could partially explain the low female proportions recorded in this study. In fact, in order to develop a *G. molesta* female-based monitoring and management approach, female attraction needs to be largely improved and female captures increased. To accomplish this, the development of new food baits or new synthetic VOC blends is needed. To date, both approaches include products of fermentation, such as terpinyl acetate and acetic acid, which have been widely investigated for *G. molesta*. For instance, the knowledge of *G. molesta* attraction to terpinyl acetate dates back almost one century, with its first use for *G. molesta* in combination with brown sugar solutions [31]. The use of these food baits has been evaluated in both MD and non-MD-treated orchards alone or in combination with other compounds, including the sex pheromone [15,16,17,18,32]. Since (*Z*)-8-dodecenyl acetate (the main component of female *G. molesta* sex pheromone) was identified [33], the possibility to replace food-baited traps using brown sugar solution plus terpinyl acetate with this sex pheromone named “orfamone” to monitor moth activity was immediately investigated [34]. After 50 years, the trend is reversed and now we are again seeking food or fermentation volatiles to enhance moth captures for both sexes. Literature offers a wide list of attractive compounds to enhance female attraction [35]; for instance, it was found that phenylacetaldehyde acts as an attractant for several lepidopteran species, and for some pests, a correlation between moth captures in traps (both males and females, thanks to the phenylacetaldehyde acting as kairomone) and subsequent damage in a cash crop was demonstrated [36]. To properly evaluate *G. molesta* attractiveness towards host plant VOCs (e.g., [10,11,12,13,14]), further field experiments are needed, considering several factors. First, compounds and blends simulating the host plants odor (e.g., pear or peach) can trigger a different response for *G. molesta* females [37]; in field experiments, for instance, total captures and female capture ratio were found to be affected by the composition and complexity of the tested blends mimicking the host plant odor [38,39,40]. Second, the geographical area can affect the result of using a specific synthetic odor [41]. Third, to transfer a new discovery into a practical innovation, the chemical stability, synthesis cost and lure longevity need to all be considered from the beginning of the volatile blend evaluation. Certainly, synthetic blends with sticky liners have to be preferred to liquid food baits for different reasons, such as the standardization of the bait and the avoidance of liquid traps use and all the relevant complications that come when servicing them [17].

## 5. Conclusions

In conclusion, the new dual-sex lure Pherocon^®^ OFM Combo Dual offers enhanced opportunities to track *G. molesta* flight, particularly in MD-treated stone fruit orchards. In our study, it was shown that this combination lure has a low impact on non-target organisms, including pollinators such as honeybees, resulting in an attraction comparable to that of the *G. molesta* sex pheromone for insect species different from *G. molesta*. Several years of field trials will be needed to develop reliable and useful intervention thresholds using this new monitoring tool. Setting the basis for this pheromone-kairomone lure usage to effectively target any insecticidal application according to the moth captures in an MD-treated orchard will also require the crop damage data. Therefore, further investigations will need to include the fruit injury evaluation in order to correlate the infestation level (expressed in terms of number of adult moth captures using this new combination lure) with the larval damage. Widening the scenario to other geographical areas and crops, like pome fruits, which in some cases are treated using MD for both *G. molesta* and *C. pomonella* and are affected by both pests at the same time, could also be beneficial for improved monitoring in the presence of MD. Finally, to use this pheromone–kairomone combination lure for management purposes, like for female removal and mass trapping, a further development of this blend is needed to increase the attraction of female moths.

## Figures and Tables

**Table 1:** Thirty-three trials of lure comparison to improve *Grapholita molesta* monitoring in peach and nectarine orchards treated and not treated with Mating Disruption (MD). Trials were performed at different crop phenological stages (according to the BBCH scale), including early fruits development (from blooming until fruits at half final size, BBCH 71-76), late fruit development (fruits increasing in size up to ripeness, BBCH 77-87) and in post-harvest (after BBCH 87).

Trial #	Site Management	Peach or Nectarine Cultivar (Estimated Harvest Date)	Trial Start Date	Crop Status During Trial	Trap Color	N° Traps per Trt.
1	MD	Romagna Red (July 3)	24 April 2018	Early fruit devel.	Orange	5
2	MD	Romagna 3000 (August 30)	24 April 2018	Early fruit devel.	Orange	5
3	MD	Romagna Big (July 19)	5 May 2018	Late fruit devel.	Orange	5
4	MD	Max 7 (September 5)	1 June 2018	Early fruit devel.	Orange	5
5	MD	August Red (August 31)	11 June 2018	Late fruit devel.	White	6
6	MD	Fercluse (July 15)	8 June 2018	Late fruit devel.	White	6
7	MD	Lami Puntoit (August 15)	8 June 2018	Late fruit devel.	White	6
8	MD	Red Late (September 1)	25 July 2018	Late fruit devel.	White	5
9	MD	Sweet Lady (August 17)	26 July 2018	Late fruit devel.	White	5
10	MD	Tardibelle (September 18)	1 June 2018	Early fruit devel.	Orange	5
11	MD	August Red (August 31)	1 June 2018	Late fruit devel.	White	5
12	MD	Romagna 3000 (August 30)	1 June 2018	Late fruit devel.	White	5
13	MD	Fairlane (September 5)	1 June 2018	Early fruit devel.	Orange	5
14	MD	Romagna 3000 (August 30)	8 March 2019	Early fruit devel.	White	5
15	MD	Romagna Big (July 19)	8 March 2019	Early fruit devel.	White	5
16	MD	Early Fresh (June 5)	8 March 2019	Early fruit devel.	White	5
17	Non-MD	Big Top (July 7)	8 March 2019	Early fruit devel.	White	5
18	Non-MD	Diamond Ray (July 22)	10 May 2019	Late fruit devel.	White	5
19	Non-MD	Ibla (August 16)	10 May 2019	Early fruit devel.	White	5
20	MD	Romagna 3000 (August 30)	10 May 2019	Late fruit devel.	White	5
21	MD	Romagna Big (July 19)	10 May 2019	Late fruit devel.	White	5
22	MD	Romagna Red (July 3)	10 May 2019	Late fruit devel.	White	5
23	MD	Early Fresh (June 5)	21 May 2019	Late fruit devel.	White	5
24	Non-MD	Big Top (July 7)	10 May 2019	Late fruit devel.	White	5
25	Non-MD	Diamond Ray (July 22)	15 July 2019	Post-Harvest	White	5
26	MD	Early Fresh (June 5)	15 July 2019	Post-Harvest	White	5
27	Non-MD	Big Top (July 7)	15 July 2019	Post-Harvest	White	5
28	MD	Romagna 3000 (August 30)	15 July 2019	Late fruit devel.	White	5
29	MD	Romagna Big (July 19)	15 July 2019	Post-Harvest	White	5
30	MD	Romagna Red (July 3)	15 July 2019	Post-Harvest	White	5
31	Non-MD	Diamond Ray (July 22)	27 August 2019	Post-Harvest	White	5
32	Non-MD	Early Fresh (June 5)	27 August 2019	Post-Harvest	White	5
33	Non-MD	Big Top (July 7)	26 August 2019	Post-Harvest	White	5

**Table 2:** Trial locations and Mating Disruption (MD) information for the sixteen peach and nectarine orchards in which the thirty-three lure comparison trials on *Grapholita molesta* monitoring were conducted. Trials from #1 to #13 were run in 2018, trials from #14 to #33 were run in 2019.

Trial #^(a)^	Cultivar (Peach or Nectarine)	Orchard Size (ha)	Geographical Coordinates (Latitude Longitude)	MD Type and Density Applied (Dispensers ha^−1^ or Dosage ha^−1^)
1, 22, 30	Romagna Red (nectarine)	1.5	44°19’14.53”N-11°51’35.67”E	ISOMATE^®^ OFM rosso FLEX at 740
2, 12, 14, 20, 28	Romagna 3000 (nectarine)	1.5	44°19’6.62”N-11°51’33.63”E	ISOMATE^®^ OFM rosso FLEX at 740
3, 15, 21, 29	Romagna Big (nectarine)	1.5	44°19’11.24”N-11°51’47.47”E	ISOMATE^®^ OFM rosso FLEX at 740
4	Max 7 (nectarine)	1.5	44°19’22.52”N-11°51’32.87”E	ISOMATE^®^ OFM rosso FLEX at 700
5	August Red (nectarine)	1.8	44°16’50.88”N-12°3’37.77”E	CheckMate OFM^®^-F (spray) at 100 ml
6	Fercluse (peach)	6.0	44°28’36.45”N-12°5’4.18”E	ISOMATE^®^ OFM rosso FLEX at 650
7	Lami Puntoit (peach)	5.0	44°28’41.19”N-12°5’7.40”E	ISOMATE^®^ OFM rosso FLEX at 650
8	Red Late (nectarine)	7.0	44°16’24.99”N-12°2’30.74”E	ISOMATE^®^ OFM rosso FLEX at 700
9	Sweet Lady (nectarine)	1.5	44°23’52.61”N-12°0’54.12”E	RAK^®^ 5+6 at 700
10	Tardibelle (peach)	4.5	44°16’46.63”N-12°2’41.86”E	Check Mate Puffer OFM at 3.5
11	August Red (nectarine)	2.8	44°17’0.08”N-12°3’24.56”E	CheckMate^®^ OFM at 350
13	Fairlane (nectarine)	2.3	44°19’40.07”N-11°52’28.81”E	RAK^®^ 5+6 at 650
16, 23, 26, 32	Early Fresh (peach)	5.4	44°19’41.32”N-11°52’25.25”E	CheckMate OFM^®^-F (spray) at 50 ml
17, 24, 27, 33	Big Top (nectarine)	1.5	44°15’50.29”N-11°53’10.73”E	Untreated with MD
18, 25, 31	Diamond Ray (nectarine)	1.5	44°19’28.13”N-11°51’12.16”E	Untreated with MD
19	Ibla (peach)	2.0	44°19’36.70”N-11°51’7.65”E	Untreated with MD

^(a)^ In trial #5 the sprayable MD was applied every 4 weeks until the end of August. In trials #16, #23 and #26, sprayable MD was applied at half dosage every 2 weeks until mid-August. Trial #32 was conducted in an orchard treated with sprayable MD until mid-August and MD application was interrupted 2 weeks before this trial start, therefore the orchard was considered as not treated with MD during the execution of trial #32.

**Table 3:** Mean (± SEM) of total *Grapholita molesta* captures over the trial period (6-8 weeks) in traps baited with Trécé Inc. lures Pherocon^®^ OFM Combo Dual (Combo Dual) and Pherocon^®^ OFM L^2^ (Pheromone). Pherocon^®^ OFM Combo Dual consisted in a grey halobutyl rubber septum loaded with the three-component sex pheromone blend for *G. molesta* and the sex pheromone of *Cydia pomonella* and a second membrane cup loaded with a blend of terpinyl acetate and acetic acid. Thirty-three trials were performed in 2018-2019 in peach and nectarine orchards treated or not treated with mating disruption (MD).

Year, Management	Mean (± SEM) *G. molesta* Captures	Lure		Statistics (glmmTMB Outputs)
		Combo Dual	Pheromone	
2018, MD	Males	7.1 ± 1.5	2.6 ± 1.0	df = 124, z = −5.687, *p* < 0.001
	Total	7.8 ± 1.6	2.6 ± 1.0	df = 124, z = −6.411, *p* < 0.001
	Proportion of females	0.08	0.00	-
2019, MD	Males	27.6 ± 6.3	0.9 ± 0.3	df = 97, z = −20.119, *p* < 0.001
	Total	28.8 ± 6.3	0.9 ± 0.3	df = 97, z = −19.923, *p* < 0.001
	Proportion of females	0.04	0.00	-
2019, non-MD	Males	46.7 ± 5.3	7.9 ± 1.9	df = 77, z = −14.277, *p* < 0.001
	Total	51.4 ± 6.0	7.9 ± 1.9	df = 77, z = −15.069, *p* < 0.001
	Proportion of females	0.09	0.00	-

Statistical analysis performed with a Generalized Linear Mixed Models using Template Model Builder (glmmTMB).

**Table 4:** Summarized outputs of the statistical analyses performed with a Generalized Linear Mixed Models using Template Model Builder (glmmTMB) to evaluate the effect of the year, Mating Disruption (MD) management, crop type (peach and nectarine) and crop phenology during trial execution (early fruit development, late fruit development and post-harvest) on *Grapholita molesta* captures in lure comparison trials (n = 33).

Explanatory Variable	Dataset Analyzed	*G. molesta* Captures	Statistics (glmmTMB Outputs)
Year (2018 *vs* 2019)	2018 MD and 2019 MD	Males	df = 232, z = 0.667, *p* = 0.505
		Total	df = 232, z = 0.736, *p* = 0.462
Management (MD *vs* non-MD)	2019 (MD and non-MD)	Males	df = 191, z = 2.083, *p* = 0.037
		Total	df = 191, z = 2.002, *p* = 0.045
Crop type (peach *vs* nectarine)	2018 MD	Males	df = 124, z = −2.250, *p* = 0.025
		Total	df = 124, z = −2.200, *p* = 0.028
Crop phenology (early *vs* late)	2018 MD	Males	df = 124, z = −0.372, *p* = 0.710
		Total	df = 124, z = −0.400, *p* = 0.690
Crop type (peach *vs* nectarine)	2019 MD	Males	df = 97, z = 1.916, *p* = 0.055
		Total	df = 97, z = 1.733, *p* = 0.083
Crop phenology (early *vs* late)	2019 MD	Males	df = 97, z = −3.965, *p* < 0.001
		Total	df = 97, z = −4.746, *p* < 0.001
Crop phenology (early *vs* post-harvest)	2019 MD	Males	df = 97, z = −1.602, *p* < 0.109
		Total	df = 97, z = −2.234, *p* < 0.026
Crop type (peach *vs* nectarine)	2019 non-MD	Males	df = 77, z = −1.621, *p* = 0.105
		Total	df = 77, z = −1.588, *p* = 0.112
Crop phenology (early *vs* late)	2019 non-MD	Males	df = 77, z = 1.258, *p* = 0.208
		Total	df = 77, z = 1.248, *p* = 0.212
Crop phenology (early *vs* post-harvest)	2019 non-MD	Males	df = 77, z = 5.800, *p* < 0.001
		Total	df = 77, z = 5.553, *p* < 0.001

**Table 5:** *Grapholita molesta* captures in ten trials of lure comparison conducted in mating disruption (MD)-treated and -not treated peach and nectarine orchards in Italy during early fruit development crop stage in 2018–2019. Mean (± SEM) of total moth captures over 6–8 weeks of trial duration. Monitoring traps were either baited with Pherocon^®^ OFM Combo Dual (Combo Dual) or Pherocon^®^ OFM L^2^ (Pheromone), both Trécé Inc. lures.

Management	Year	Trial #	Lure Type	*G. molesta* Captures		Unpaired Two-Sample Test on Male Captures
				Females	Males	
MD	2018	1	Combo Dual	0.8 ± 0.5	3.2 ± 0.7	t = 2.810, df = 8, *p* = 0.023
			Pheromone	0.0 ± 0.0	0.8 ± 0.5	
		2	Combo Dual	4.6 ± 1.1	31.6 ± 9.9	t = 1.140, df = 8, *p* = 0.287
			Pheromone	0.0 ± 0.0	18.8 ± 10.9	
		4	Combo Dual	0.2 ± 0.2	3.4 ± 0.9	-
			Pheromone	0.0 ± 0.0	0.0 ± 0.0	
		10	Combo Dual	0.4 ± 0.2	1.4 ± 0.9	W = 16, *p* = 0.441
			Pheromone	0.0 ± 0.0	0.4 ± 0.4	
		13	Combo Dual	0.4 ± 0.2	0.2 ± 0.2	-
			Pheromone	0.0 ± 0.0	0.0 ± 0.0	
	2019	14	Combo Dual	1.2 ± 0.4	89.2 ± 16.3	t = 8.647, df = 8, *p* < 0.001
			Pheromone	0.0 ± 0.0	2.0 ± 0.6	
		15	Combo Dual	1.2 ± 0.5	11.6 ± 1.1	-
			Pheromone	0.0 ± 0.0	0.0 ± 0.0	
		16	Combo Dual	3.0 ± 0.5	147.0 ± 4.1	W = 25, *p* = 0.012
			Pheromone	0.0 ± 0.0	6.6 ± 2.0	
non-MD	2019	17	Combo Dual	0.6 ± 0.4	21.6 ± 3.7	W = 25, *p* = 0.010
			Pheromone	0.0 ± 0.0	2.8 ± 0.2	
		19	Combo Dual	0.0 ± 0.0	8.0 ± 2.0	-
			Pheromone	0.0 ± 0.0	0.0 ± 0.0	

Unpaired two-sample T-test was used to compare means of data normally distributed: #1, #2, #14. Non-parametric unpaired two-sample Mann–Whitney U test was used to analyze data not normally distributed: #10, #16, #17.

**Table 6:** *Grapholita molesta* captures in fifteen trials of lure comparison conducted in mating disruption (MD)-treated and -not treated peach and nectarine orchards in Italy during late fruit development crop stage in 2018–2019. Mean (± SEM) of total moth captures over 6–8 weeks of trial duration. Monitoring traps were either baited with Pherocon^®^ OFM Combo Dual (Combo Dual) or Pherocon^®^ OFM L^2^ (Pheromone), both Trécé Inc. lures.

Management	Year	Trial #	Lure Type	*G. molesta* Captures		Unpaired Two-Sample Test on Male Captures
				Females	Males	
MD	2018	3	Combo Dual	0.2 ± 0.2	3.2 ± 1.4	t = 1.201, df = 8, *p* = 0.264
			Pheromone	0.0 ± 0.0	1.2 ± 0.6	
		5	Combo Dual	0.5 ± 0.3	7.5 ± 2.4	t = 3.114, df = 10, *p* = 0.011
			Pheromone	0.0 ± 0.0	1.5 ± 1.1	
		6	Combo Dual	0.0 ± 0.0	0.3 ± 0.2	-
			Pheromone	0.0 ± 0.0	0.0 ± 0.0	
		7	Combo Dual	0.3 ± 0.2	0.0 ± 0.0	-
			Pheromone	0.0 ± 0.0	0.0 ± 0.0	
		8	Combo Dual	0.2 ± 0.2	0.4 ± 0.2	-
			Pheromone	0.0 ± 0.0	0.0 ± 0.0	
		9	Combo Dual	1.0 ± 0.5	17.2 ± 4.6	t = 1.927, df = 8, *p* = 0.090
			Pheromone	0.0 ± 0.0	7.2 ± 2.3	
		11	Combo Dual	0.0 ± 0.0	26.0 ± 6.3	t = 3.193, df = 8, *p* = 0.013
			Pheromone	0.0 ± 0.0	5.4 ± 4.9	
		12	Combo Dual	0.0 ± 0.0	1.0 ± 0.5	W = 18, *p* = 0.232
			Pheromone	0.0 ± 0.0	0.2 ± 0.2	
MD	2019	20	Combo Dual	0.2 ± 0.2	11.0 ± 1.3	W = 25, *p* = 0.009
			Pheromone	0.0 ± 0.0	0.6 ± 0.6	
		21	Combo Dual	0.6 ± 0.4	4.2 ± 1.1	t = 5.014, df = 8, *p* = 0.001
			Pheromone	0.0 ± 0.0	0.2 ± 0.2	
		22	Combo Dual	0.2 ± 0.2	2.8 ± 0.4	-
			Pheromone	0.0 ± 0.0	0.0 ± 0.0	
		23	Combo Dual	0.4 ± 0.2	3.6 ± 0.2	-
			Pheromone	0.0 ± 0.0	0.0 ± 0.0	
		28	Combo Dual	1.4 ± 0.7	1.8 ± 0.6	-
			Pheromone	0.0 ± 0.0	0.0 ± 0.0	
non-MD	2019	18	Combo Dual	0.0 ± 0.0	7.4 ± 1.8	t = 6.358, df = 8, *p* < 0.001
			Pheromone	0.0 ± 0.0	0.2 ± 0.2	
		24	Combo Dual	7.2 ± 2.4	43.6 ± 7.4	t = 5.627, df = 8, *p* < 0.001
			Pheromone	0.0 ± 0.0	7.2 ± 2.4	

Unpaired two-sample T-test was used to compare means of data normally distributed: #3, #5, #9, #11, #21, #18, #24. Non-parametric unpaired two-sample Mann–Whitney U test was used to analyze data not normally distributed: #12, #20.

**Table 7:** *Grapholita molesta* captures in eight trials of lure comparison conducted in mating disruption (MD)-treated and -not treated peach and nectarine orchards in Italy during post-harvest in 2019. Mean (± SEM) of total moth captures over 6–8 weeks of trial duration. Monitoring traps were either baited with Pherocon^®^ OFM Combo Dual (Combo Dual) or Pherocon^®^ OFM L^2^ (Pheromone), both Trécé Inc. lures.

Management	Year	Trial #	Lure Type	*G. molesta* Captures		Unpaired Two-Sample Test on Male Captures
				Females	Males	
MD	2019	26	Combo Dual	1.2 ± 0.6	21.2 ± 3.2	t = 9.678, df = 8, *p* < 0.001
			Pheromone	0.0 ± 0.0	0.4 ± 0.2	
		29	Combo Dual	3.2 ± 1.5	5.8 ± 1.8	-
			Pheromone	0.0 ± 0.0	0.0 ± 0.0	
		30	Combo Dual	0.8 ± 0.4	5.6 ± 1.7	-
			Pheromone	0.0 ± 0.0	0.0 ± 0.0	
non-MD	2019	25	Combo Dual	3.0 ± 1.3	52.4 ± 4.3	W = 25, *p* = 0.012
			Pheromone	0.0 ± 0.0	0.8 ± 0.4	
		27	Combo Dual	17.6 ± 1.7	99.8 ± 5.0	t = 9.042, df = 8, *p* < 0.001
			Pheromone	0.0 ± 0.0	32.8 ± 4.7	
		31	Combo Dual	6.6 ± 1.9	80.2 ± 16.3	t = 7.499, df = 8, *p* < 0.001
			Pheromone	0.0 ± 0.0	2.8 ± 0.9	
		32	Combo Dual	2.4 ± 0.7	25.4 ± 4.4	t = 7.340, df = 8, *p* < 0.001
			Pheromone	0.0 ± 0.0	1.2 ± 0.5	
		33	Combo Dual	5.0 ± 1.9	81.6 ± 9.1	t = 5.485, df = 8, *p* = 0.001
			Pheromone	0.0 ± 0.0	23.4 ± 6.0	

Unpaired two-sample T-test was used to compare means of data normally distributed: #26, #27, #31, #32, #33. Non-parametric unpaired two-sample Mann–Whitney U test was used to analyze data not normally distributed: #25.
